# Trauma-Induced Damage-Associated Molecular Patterns-Mediated Remote Organ Injury and Immunosuppression in the Acutely Ill Patient

**DOI:** 10.3389/fimmu.2018.01330

**Published:** 2018-06-15

**Authors:** Mickael Vourc’h, Antoine Roquilly, Karim Asehnoune

**Affiliations:** ^1^Laboratoire UPRES EA3826 “Thérapeutiques cliniques et expérimentales des infections”, IRS2 – Nantes Biotech, Université de Nantes, Nantes, France; ^2^Intensive Care Unit, Anesthesia and Critical Care Department, Hôtel Dieu, University Hospital of Nantes, Nantes, France

**Keywords:** damage-associated molecular pattern, posttraumatic immunosuppression, remote organ dysfunction, hospital-acquired infection, critical care, transfusion

## Abstract

Trauma is one of the leading causes of death and disability in the world. Multiple trauma or isolated traumatic brain injury are both indicative of human tissue damage. In the early phase after trauma, damage-associated molecular patterns (DAMPs) are released and give rise to sterile systemic inflammatory response syndrome (SIRS) and organ failure. Later, protracted inflammation following sepsis will favor hospital-acquired infection and will worsen patient’s outcome through immunosuppression. Throughout medical care or surgical procedures, severe trauma patients will be subjected to endogenous or exogenous DAMPs. In this review, we summarize the current knowledge regarding DAMP-mediated SIRS or immunosuppression and the clinical consequences in terms of organ failure and infections.

## Introduction

Trauma represents the most extreme form of human tissue damage and is one of the leading causes of mortality and disabilities in the world (1). Ischemia-reperfusion, crush syndrome, surgical procedures, acidosis, hypoxemia, blood loss, or massive transfusion can all give rise to secondary tissue damage. Following trauma, a number of mediators, also called damage-associated molecular patterns (DAMPs), are released in the bloodstream by injured tissues. The recognition of DAMPs by immune cells initiates a systemic inflammatory response syndrome (SIRS) that induces physiological changes such as hypo or hyperthermia, elevated heart rate and leukocytosis/leukocytopenia (2). Early uncontrolled and protracted SIRS has been reported to be a risk factor of “sterile” organ failure (3) and of delayed hospital-acquired infection (mainly pneumonia) (3, 4). The high susceptibility to secondary infection after the initial phase of sepsis or trauma is attributable to an intense and long-term compensatory anti-inflammatory response (CARS) (5–7) leading to posttraumatic immunosuppression (IS). Initially, IS corresponds to a homeostatic phenomenon to avoid the remote organ injuries caused by the initial SIRS. It becomes deleterious when it persists, rendering patients prone to secondary bacterial (8) or fungal infections and viral reactivation.

Damage-associated molecular patterns are the origins of both SIRS and IS and are, therefore, promising targets as biomarkers for patient stratification and as therapeutic targets.

## DAMPs Definition

Damage-associated molecular patterns are endogen nuclear, mitochondrial, or cytosolic molecules that have physiological functions inside the cell. They activate innate and adaptive immunity when released into the extracellular milieu. Innate immunity cells, mainly antigen-presenting cells (APC) such as dendritic cells (DCs) and neutrophils (PMNs) recognize DAMPs *via* pattern recognition receptors (PRRs). After PRR activation, PMNs and APC give rise to the local production of cytokines, chemokines, and other soluble factors. Local inflammatory response aims to ensure adequate tissue repair and may also generate a systemic and uncontrolled inflammatory response inducing remote organ failure.

Damage-associated molecular patterns released after trauma are also called alarmins. Since any molecule expelled in the microenvironment after tissue damage may be considered as an alarmin, it is of major importance to detect those that are clinically relevant and immunologically active. Clinically pertinent alarmins during trauma were defined in a consensus in 2006 (9) as substances:

- immediately released after trauma,
- responsible for immune cell activation whose concentration reflects the severity of trauma,
- giving rise to pro-inflammatory response on cultured cells with a clearly elucidated mechanism of activation,
- with plasma levels that correlate with the extent of the inflammatory response.

Finally, trauma alarmins have redundant activity on several receptors with highly variable effects depending on the microenvironment (10).

## Characterization of DAMPs Released After Trauma

### Nucleic Acids

All human cells contain nucleic acids [desoxynucleic acid (DNA) or messenger RNA] (11) or mitochondrial DNA (mtDNA) and mature erythrocytes may also retain some residual non-functional mtDNA (12). During severe trauma, nuclear and mitochondrial nucleic acids are released into the cytosol and in the bloodstream. The plasma levels of DNA increase parallel to the severity of the trauma and the concentration of mtDNA is correlated with the occurrence of acute lung injury (ALI), the severity of SIRS and the occurrence of multiple organ dysfunction syndrome (MODS) (13–17). Nuclear DNA recognition by monocytes triggers the same inflammatory response as microbial nucleic acids (also called PAMPs or pathogen-associated molecular pattern). In particular, monocytes produce IL-6 after stimulation by nuclear DNA (18) or IL-8 after exposition to messenger RNA (19).

### High-Mobility Group Box 1 (HMGB1)

High-mobility group box 1 is a nuclear chaperone protein that regulates DNA transcription. In physiological settings, HMGB1 binds to DNA and bends it to facilitate gene transcription. After severe trauma, HMGB1 is either secreted by activated or stressed immune and non-immune cells or can leak out from dead cells (20). In the same way, hypoxia or ischemia-reperfusion in hepatocytes or cardiomyocytes cause time-dependent extracellular release of HMGB1 (21, 22). Its plasma concentration peaks within 6 h after injury and the concentration remains elevated for at least 24 h (23). Its level 30 min after trauma correlates with the injury severity score (ISS), SIRS, MODS, death, and with the amplitude of immune activation (24, 25). For example, a high level of HMGB1 after trauma was associated with lung dysfunction and longer duration of mechanical ventilation (26). Moreover, HMGB1 release after trauma with bone fracture can exert remote effects on several organs. For example, HMGB1 can worsen cerebral lesion after ischemic brain injury (BI) (27) or lead to lung injury (28). Interestingly, the variations of redox conditions influence HMGB1 activity. HMGB1 contains two redox sensitive sites that deeply impact its function (29). During severe trauma, excessive production of radical oxygen species enhances oxidative stress and leads to multiple redox reactions. HMGB1 function shifts to promote severe inflammation when oxidative stress increased (30). At the opposite, the reduced form rather enhances chemotactic signaling. Finally, this protein has a poor pro-inflammatory activity but acts as a cofactor of inflammation with lipopolysaccharide (LPS), nuclear DNA, or IL-1β.

### Heat-Shock Proteins (HSPs)

Heat-shock proteins are molecular chaperones that control intracellular trafficking and prevent the misfolding of polypeptide chains. These proteins are named according to their molecular weight and are expressed both constitutively and under stressful conditions (31). HSPs are present in the bloodstream of healthy volunteers (32). Their circulating levels decrease with aging (33) and increase under several pathological conditions. HSPs are released into the extracellular compartment after trauma (34–36). In particular, in severe trauma with an ISS score higher than 16, HSP72 levels were significantly higher compared with healthy controls (37). Interestingly, severe trauma patients with the highest HSP72 circulating levels on hospital admission had better survival rates (37). HSPs activate both innate and adaptive immunity and can trigger pro-inflammatory response. In particular, HSPs promote antigen presentation and the maturation of DCs as demonstrated by the upregulation of the major histocompatibility complex (MHC) class II molecules (38) and co-stimulatory signals, such as CD80 and CD86 (39, 40).

### S100 Proteins

This family includes more than 20 distinct proteins. S100 proteins are calcium-binding proteins and are mainly expressed by myeloid cells. Their release in extracellular compartments is secondary to cell damage or phagocytosis (41). Three S100 proteins are specifically linked to innate immune functions: S100A8, S100A9, and S100A12. Protein S100A8, also called Calgranulin A or Myeloid-related protein 8 (MRP8), and S100A9, also called Calgranulin B or MRP14 are found in monocytes and macrophages (42). Protein S100A12 (Calgranulin C) is mostly expressed in granulocytes (43). During inflammatory response, monocytes or macrophages release MRP8 (S100A8) and MRP14 (S100A9) into the circulation. They then form a heterodimer (44) and are recognized by PRRs. After severe burn injury, myeloid-related protein (MRP) levels in bloodstream are correlated with poor outcome (45). S100β protein is specifically released after acute BI (46, 47). Similar to HMGB1, MRP8 and MRP14 levels in the bloodstream increase early in the acute phase of trauma or BI (48).

## DAMPs Activate Several Receptors, Signaling Pathways, and Cellular Subsets

**Figure 1:**
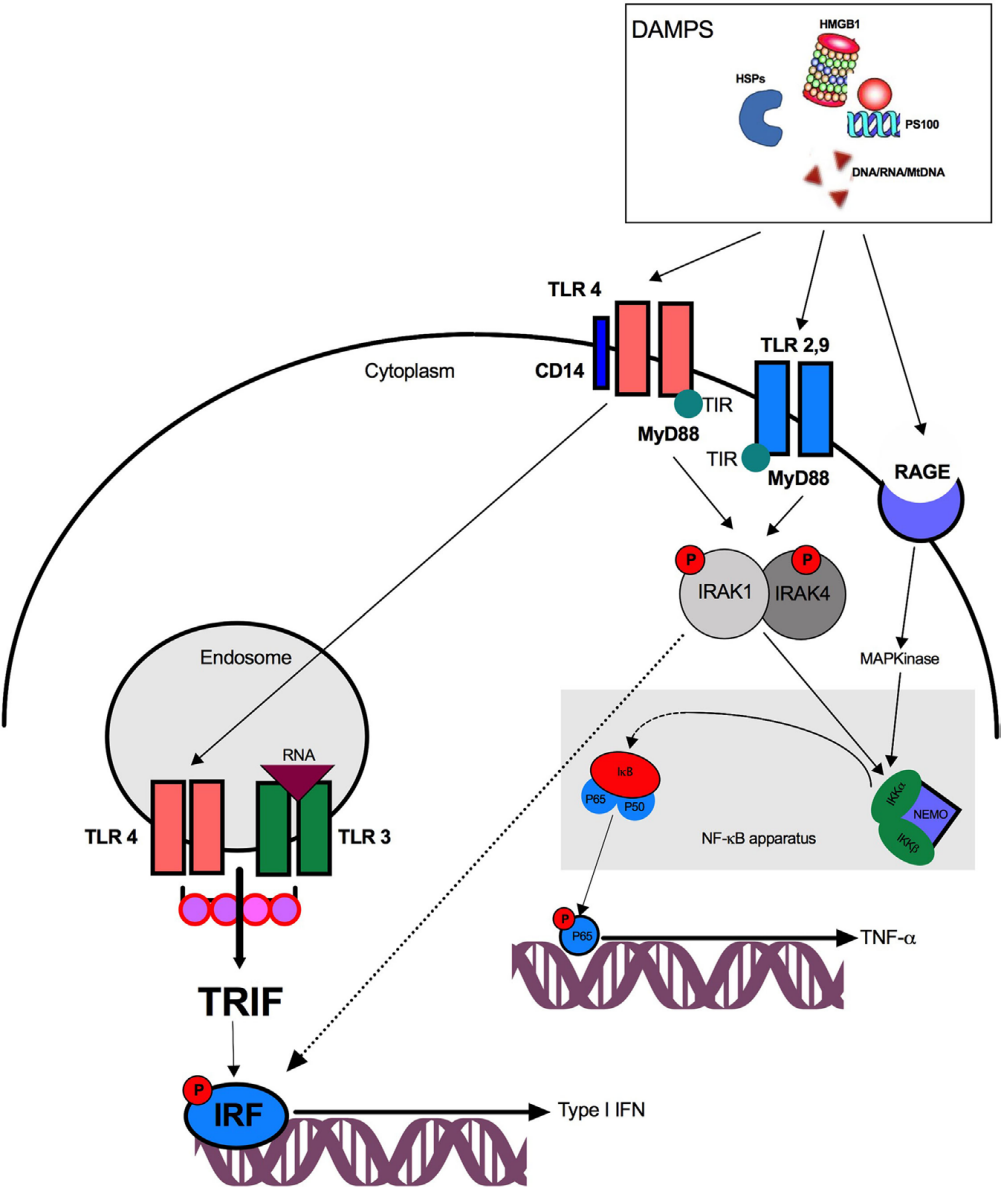
Pathway activation after damage-associated molecular patterns (DAMPs) stimulation. DAMPs are recognized by surface or endosomal TLRs and trigger either TRIF and NF-κB activation. TLR4, toll-like receptor 4; TIR, toll/interleukin-1 receptor; DNA, desoxyribonucleic acid; RNA, ribonucleic acid; IRF, interferon regulatory factor; TRIF, TIR domain-containing adaptor protein inducing interferon β; NF-κB, nuclear factor κB; NEMO, NF-κB essential modulator; IRAK, IL-1 receptor-associated kinase-4; MyD88, myeloid differentiation protein 88; RAGE, receptor for advanced glycation end products; HMGB1, high-mobility group box 1; HSP, heat-shock protein; IKK, Iκ-B kinase.

Damage-associated molecular patterns released by injured organs, tissues, or cells can be detected by several receptors including PRRs and activate several pathways (Figure 1). Of interest, a given DAMP may stimulate several receptors and engage different pathways—a phenomenon called receptor redundancy.

### Pathogen Recognition Receptors

Pattern recognition receptors are expressed by a large variety of innate immune cells including PMNs, natural killer lymphocytes (NK), macrophages and DCs. Among PRRs, the main receptors involved after trauma are nucleotide oligomerization domain (NOD)-like receptors (NLRs) and toll-like receptors (TLRs). NLRs are intracellular PRRs that are localized in the cytoplasm. They usually sense degradation products from bacterial cell wall components. NOD receptors activate the nuclear transcription factor NF-κB and the MAP kinases leading to pro-inflammatory cytokine production.

Toll-like receptors, initially described for PAMP recognition, also play a key role in the recognition of DAMPs such as: nucleic acids, HMGB1, HSPs, and S100 proteins. TLRs are localized on the cell surface and/or within the intracellular endosomal compartment. After DAMPs binding, TLRs dimerize and recruit adapter proteins through their intracellular TIRs (Toll/Interleukin-1 receptor) domains. All TLRs except TLR3 signal through the adapter myeloid differentiation protein 88 (MyD88). Downstream, and depending on the TLR understudy, MyD88 can activate two distinct signaling pathways. The first requires IL-1 receptor-associated kinase (IRAK) recruitment which activates the NF-κB apparatus and leads to TNF-α, IL-6, and IL-8 production. The second involves TIR domain-containing adaptor protein inducing interferon β (TRIF) activation that induces interferon regulatory factor (IRF) phosphorylation allowing the synthesis of type I interferon (IFN) (49, 50). TLR4 plays a singular role in DAMP detection and (51) activates either NF-κB *via* MyD88–IRAK or TRIF after TLR4 translocation to the endosome through a CD14-dependent mechanism. Interestingly, TLR3 exclusively triggers the TRIF-dependent pathway.

The IRF pathway leads to type I IFN production and is critical for human leukocyte antigen (HLA)-DR regulation and co-stimulatory molecules (CD80/CD86) expression on APCs. The NF-κB pathway leads to the production of several pro-inflammatory mediators (including TNF-α) and may be activated by extracellular signals present in the blood and organs after trauma, such as reactive oxygen species, cytokines, and complement fragments (52). NF-κB activation requires a complex of kinases which are called IκB Kinase (IKK). IKK induces the degradation (phosphorylation and ubiquitination) of the cytoplasmic inhibitor of NF-κB: IκB-α. This degradation enables the translocation of NF-κB sub-units into the nucleus. After binding to the promoter of the gene, the heterodimer form of NF-κB (P65/P50) enhances whereas the homodimer P50/P50 inhibits the transcription of proinflammatory cytokines. NF-κB activation also gives rise to inflammasome formation and caspase-1 release and promotes IL-1β synthesis (53).

Nucleic acids trigger TLR3 (mRNA) or TLR9 (DNA and mtDNA) activation. Among mitochondrial damage-associated molecular patterns, N-formyl peptides (F-MIT), and mtDNA can give rise to sterile inflammation. The former activates formyl peptide receptor-1 (FRP1) and the latter binds TLR9 and promotes the formation of NOD-like receptor pyrin domain containing-3 (NLRP3) inflammasome (13, 54).

High-mobility group box 1 activates NF-κB transcription factor through TLR2, TLR4, or TLR9 and leads to TNF-α, IL-1β, and IL-6 production (55–57). The recognition of HMGB1 by TLR4 requires myeloid differentiation factor 2 (58). HMGB1 can also link to LPS, thereby strengthening its ability to activate TLR4 through CD14 (59). Finally, HMGB1 can complex with CpG-ODN and bind TLR9 to enhance cytokine production of APCs (60).

Heat-shock proteins promote antigen presentation, the maturation of DCs and activate the TLR-MyD88-NF-κB pathway (38). MRP8 (PS100A8), MRP14 (PS100A9), and PS100A12 are recognized by TLR4 or TLR2 (45, 61).

### Receptor for Advanced Glycation End Product (RAGE) and Triggering Receptor Expressed on Myeloid Cells-1 (TREM-1)

Triggering receptor expressed on myeloid cells-1 is a member of the immunoglobulin superfamily expressed on monocytes and PMNs that synergize with TLR4 to mediate the effects of HMGB1 (62). TREM-1 is associated with an immunoreceptor tyrosine-based activation motif and an adaptor molecule called DAP12 for signal transduction. *In fine*, TREM-1 pathway phosphorylates extracellular signal-regulated kinase and phosphoinositide-3 kinase leading to NF-κB activation (63). HMGB1 is also detected by the RAGE (64), an immunoglobulin superfamily cell surface receptor. Contrary to TLRs, RAGE is a specific receptor for DAMP that is not activated by PAMP. It activates NF-κB pathway through RAS family proteins and MAP kinase phosphorylation (65). Finally, among S100 proteins, RAGE recognize S100A12 and S100β (46, 47) and subsequently engage the NF-κB pathway.

Overall, DAMPs triggers massive cytokine relapse including TNF-α, IL-1, IL-6, IL-8, IL-12 and IFN types I and II. These mediators amplify the activation, maturation, proliferation, and recruitment of immune cells at the site of trauma, causing indirect activation of innate and adaptive immune cells such as DCs or T cells (66).

### DAMPs Activate Neutrophils and DCs

During severe trauma, F-MIT activate PMNs *via* FRP1, a G protein-coupled surface receptors (GPCR) (67) and trigger their chemotaxis and phagocytosis (68). In parallel, HMGB1/RAGE enhances PMNs recruitment toward injured tissues and amplifies the inflammatory response *via* NF-κB transcription factor activation (69). HMGB1 can also activate PMNs through TLR4 or TLR7 during ischemia-reperfusion injury (70). Besides, extracellular adenosine 5’-triphosphate (ATP) release enhances PMNs adhesion to blood vessel wall upon sterile injury (71) and promotes cell migration to injury site (72). Finally, PMNs activation contributes to SIRS, cardiovascular collapse, and remote organ injury (73). Notably, the release of metalloproteinase 9 (MMP9) after traumatic brain injury (TBI) is involved in secondary brain damage (74).

Damage-associated molecular patterns will also activate DCs and trigger both the innate and the adaptive immune response (75). Among the most described DAMPs, activation and maturation of DCs are mainly driven by HMGB1 binding to TLR2, TLR4 (55), or RAGE (76, 77). HMGB1 also acts as a chemokine for DCs when binding to CXCR4. Then, HSPs bind TLR4 (78) and participate in antigen processing by DC before presentation (79) to T cells. Moreover, mtDNA and especially mitochondrial transcription factor A can synergize with CpG to induce type I IFN release (80, 81). DCs also recognize extracellular ATP by high affinity purinergic receptors P2Z/P2X7 (82) leading to inflammasome activation (83).

## Novel Insights

### DAMPs Participate in Posttraumatic IS

Following trauma, DAMPs participate in IS that renders patients prone to secondary infections. Timmermans et al. recently evaluated the role of DAMPs in IS in 166 adult trauma patients. The authors reported that plasma nuclear DNA and HSP70 levels correlated negatively with HLA-DR expression. Moreover, higher levels of circulating nuclear DNA and a further decrease in HLA-DR expression on blood monocytes were associated with infections. Overall, the role of DAMPs in the induction of an overwhelming inflammatory process leading to remote organ failure is well known. This study is one of the first descriptions that associates plasma levels of DAMPs with IS and with secondary infections (34).

### DAMPs Induce Endotoxin Tolerance

After massive DAMP release, the early overwhelming inflammatory response can further leave an immunological scar that will be responsible for protracted immune alterations (IS) (84) and will worsen patient outcome. This IS decreases the capacity of monocytes to produce inflammatory cytokines after TLR stimulation (52, 85, 86) and APC to prime antigen on type 2 MHC molecules. Austermann et al. highlighted that MRP linkage with TLRs induced phagocyte hyporesponsiveness to subsequent TLR agonist stimulation and was correlated with poor outcome (45). In the same way, HSPs can also induce a tolerogenic response. After HSP70 recognition by monocytes through CD14/TLR4 complex, subsequent LPS stimulation fails to activate IKK. As a result, the phosphorylation of P65 is altered and prevents the NF-κB activation (35).

Damage-associated molecular patterns may also induce epigenetic alterations including chromatin modifications leading to a transient silencing of the transcription of pro-inflammatory mediators (87). These epigenetic modifications induce hyporesponsiveness (tolerance) to subsequent stimulation of TLRs. After trauma, Austermann et al. reported that after TLR–MyD88–NF-κB activation by MRPs, RelB nuclear translocation increases the methylation of the promoter gene of TNF-α and reduces its transcription (45).

Finally, DAMPs release in severe trauma patients can induce tolerance similarly to post-septic scenarios (52). After trauma, although early tolerance attenuates the severity of the organ failures caused by ischemia-reperfusion, long-term tolerance can also increase susceptibility to secondary infection.

### Immunosuppressive Properties of HMGB1

The rate of infections after severe TBI is very high—up to 40%—and is largely correlated with IS (8). TBI-induced IS consists in monocytic deactivation along with decreased expression of HLA-DR on monocytes and lymphopenia (88–90). In TBI patients, our team confirmed that the downregulation of HLA-DR on monocytes leads to an increased susceptibility to secondary infection (91). At the same time, cytotoxic response of NK cells is altered (91), CD4^+^Th1-type cell response is suppressed (92) whereas Th2-type response is enhanced (93). As a result, TBI appears to affect both innate and adaptive immunity.

A strong correlation between the severity of BI and the intensity of immune depression has been previously reported (94). It is generally believed that the autonomic nervous system plays a major role in IS after BI because catecholamines are elevated and alter immunity. However, recent clinical (95) and experimental (96) studies have challenged this paradigm and there is evidence that alarmins are a cornerstone of IS after TBI.

High-mobility group box 1 is released in the early phase after BI and has been identified as a key player in overwhelming sterile inflammatory response (97). HMGB1 can also trigger the proliferation of suppressive cells (89, 90). For example, after BI HMGB1 induces the expansion of altered monocytes in the spleen. This was characterized by a low level of type II MHC molecule expression and reduced cytokine production (TNF-α and IL-12) in response to TLR agonists (97). These altered monocytes can suppress lymphocytes and lead to lymphopenia, a hallmark of IS after BI.

Finally, HMGB1 may alter the prognosis of severe BI patients not only through the initial pro-inflammatory response but also because it may induce IS which is a major cause of secondary bacterial infections.

### DAMPs Can Induce IS Without Prior Overwhelming Inflammatory Response

**Figure 2:**
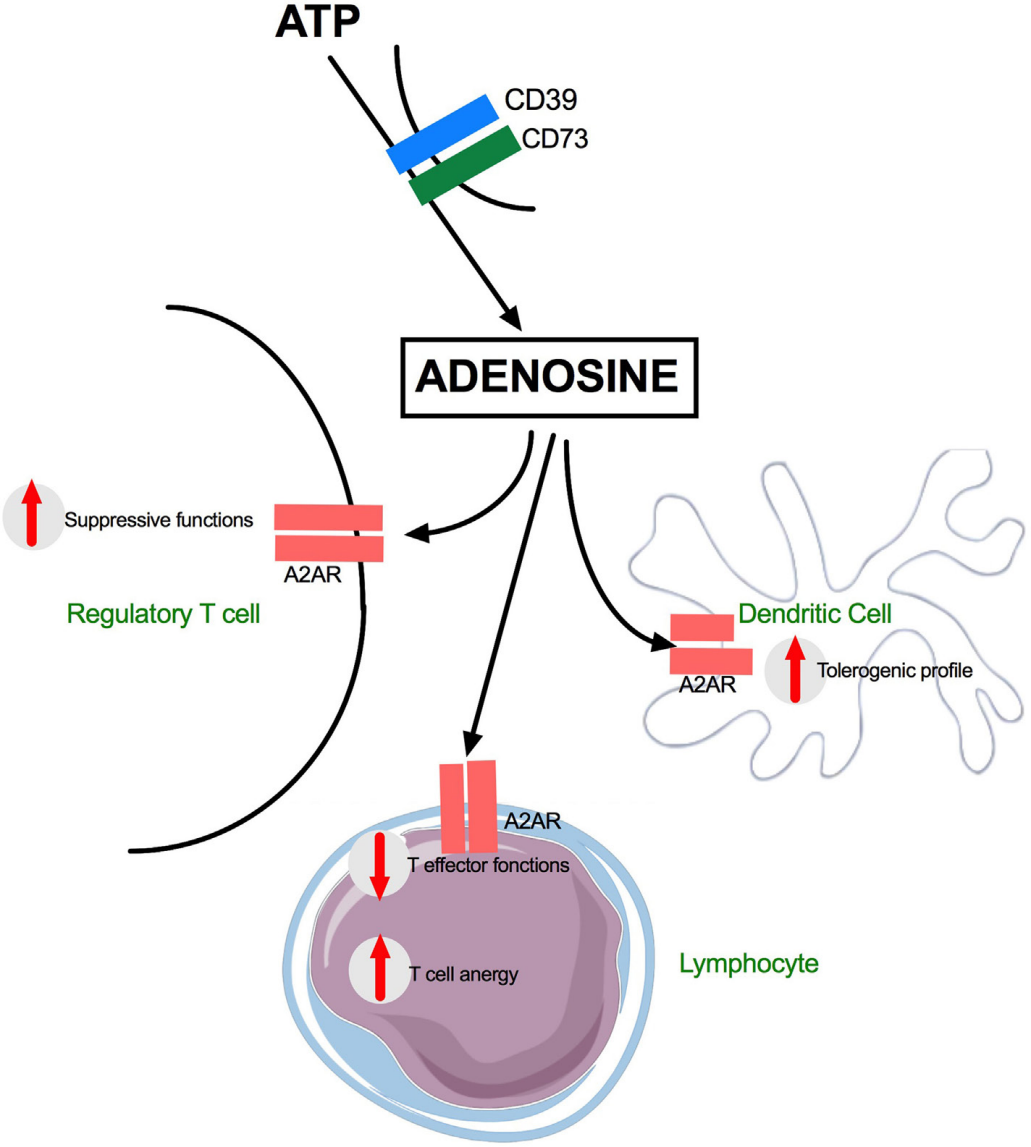
Adenosine effects on dendritic and T cells during trauma. G protein-coupled surface receptors (GPCR) are key actors of Adenosine mediated inflammatory after trauma. A2AR receptors are expressed on many subsets including Regulatory T cells, effector T cells, and dendritic cells. ATP, adenosine 5’-triphosphate; CD39, nucleoside triphosphate diphosphohydrolase-1; CD73, surface-expressed ecto-5′-nucleotidase; A2AR, adenosine A2A receptor.

Recently published data suggest that DAMPs may directly induce IS without the need for a first overwhelming inflammatory response (98). Endogenous purine nucleosides are major regulators of the inflammatory response. In this setting, adenosine, which is a catabolite of ATP, signals through the binding and activation of GPCR such as A2AR, a purinergic receptor. High amount of adenosine released after cell injuries including severe trauma may induce a major inflammatory response. More recently, it was shown that adenosine also promotes Th2 immunity, a critical component of posttraumatic IS. Indeed, when binding to GPCR, adenosine may activate DCs to promote the production of IL-4 by CD4 T cells (99, 100). In the study by Patel et al. (98), the authors suggested that the specific GPCR for adenosine (A_2_AR) initiates Th2 immune response. In their model, A_2_AR induced expression of the alarmin IL-33, which subsequently triggered ILC-2 (Type 2 Innate lymphoid cells) and Th2 cell activation and production of IL-4 and IL-13—cytokines which are known to participate in posttraumatic IS. The results of Patel et al. are important because they suggest that a single DAMP such as adenosine may directly induce a Th2 response. There is another way by which endogenous purine molecules may alter immunity. The release of ATP from necrotic cells is increased in inflamed tissues like after severe trauma. This alarmin activates DCs and cellular immunity in general *via* P2 receptors (99–101). CD39 degrades ATP to adenosine (a trigger of the Th2 response, see above), and CD39 expression on CD4^+^Foxp3^+^ Treg is known to contribute to the suppressive functions of these cells (102–104). This is important since CD39 is constitutionally expressed on Treg cells and is a critical pathway for negative regulation of inflammation (Figure 2).

Overall, these data show that alarmins play a role in the production of IL-4 which is mainly produced by activated T cells. The antagonistic nature of IL-4 on Th1 response makes it an important potential factor in IS. Activated Treg (CD39^+^) further alters immunity by increasing the levels of Adenosine.

### Immune Dysfunction Following Transfusion Is Triggered by DAMP

Transfusion is reported to suppress innate immunity (105) and is associated with an increased risk of death during surgery. Multiple trauma patients often require massive transfusion (106) and transfusion correlates with infection in a dose-dependent manner (107, 108). Packed red blood cells (PRBC) contain immunologically active compounds. Several studies have reported changes in supernatant composition during storage of red blood cell (RBC) and hypothesized that these modifications could explain morbidity (109, 110). However, large randomized studies have assessed the impact of the storage duration of PRBC in blood banks and provided conflicting results regarding patient outcome (111–113). Up to 25% of RBC can undergo hemolysis within 24 h following transfusion regardless of group incompatibility or immunologic reactions (114). RBC contains most of the DAMPs described above. As a result, hemolyzed RBC release high amounts of DAMPs into the circulation including Heme, HSP70, Retinol-Binding Protein-4 (RBP-4), IL-33, S100 proteins, and adenosine (115). Heme acts as a DAMP and promotes the formation of the inflammasome in LPS-primed macrophages after TLR4 stimulation (116, 117). RBP4, which was recently identified in PRBC (118), is a TLR4 agonist and can be considered as a DAMP (119). Most RBC DAMPs will trigger NF-κB or IRF activation leading to TNF-α or type I IFN production influencing cell survival, differentiation, and proliferation (120). RBC lysis has also been reported to be responsible for IL-33 relapse (115). IL-33 is a nuclear-associated IL-1 family cytokine inducing type 2 cytokine response (121) and promoting regulatory T-cell response (122). ATP intracellular concentration in RBC is very high (123). After extracellular release, ATP is cleaved in adenosine which also acts as a DAMP (see above). Adenosine either triggers inflammatory response through NLRP3 activation and inflammasome formation or promotes Th2 response though GPCR activation (121). RBC lysis can release HSP70 (115) which stimulates monocytes and DCs *via* TLR2 and TLR4/CD14 pathways. This DAMP could account for transfusion-related acute renal failure (124). Finally, non-functional mtDNA can be retained in mature RBC and participate in the sterile inflammation response after transfusion *via* TLR9 stimulation and NLRP3 inflammasome formation.

Our team is conducting a prospective study on transfusion-induced immunomodulation and organ failure with the aim to identify DAMPs in the supernatant of labile blood products (NCT02763410). Our preliminary results have shown that the composition of each PRBC is highly variable even between 2 PRBC with the same storage duration. Our results may, therefore, account for controversial results in the last large randomized studies. For example, we have noted major differences in terms of RBP-4 or SDF1-α, stromal cell-derived factor 1 (CXCL12) concentrations (unpublished data), see Figures S1A,B in Supplementary Material. Even if CXCL12 is not strictly a DAMP, it can complex and synergize with HMGB1 and enhance CXCR4 activation (125). Finally, further studies are required to focus on RBC supernatant composition to study patient outcome and to better delineate the exact role of the DAMPs that are released after blood transfusion.

## Clinical Consequences: Damp-Related Organ Dysfunction

The ISS predicts further organ failure and patient outcome (126). MODS incidence can reach 60% of patients (127). If DAMP-induced remote organ dysfunction is now a widely accepted phenomenon, its exact pathophysiology is only partially understood. Organ dysfunction after trauma follows a biphasic pattern. Early MODS is usually driven by an uncontrolled SIRS, and late MODS involves IS-induced nosocomial infections (mainly pneumonia).

### Early MODS

Despite well-conducted resuscitation, hypotension, transfusion support and the combined effects of rhabdomyolysis, ischemia-reperfusion, acidosis, anemia, hypoxemia, and hypovolemia induce cell injuries leading to remote organ dysfunction [i.e., acute renal failure, disseminated intravascular coagulation (DIC), or ALI]. After cell injuries, massive DAMPs release leads to SIRS and when the response is overwhelming, the risk of MODS is high. The role of DAMPs in the occurrence of MODS is illustrated by the high level of circulating HMGB1 after trauma which has been associated with the magnitude of SIRS, MODS and death (24, 128, 129). Moreover, a high amount of HMGB1 in patient serum will give rise to lung injury characterized by tight junction alteration and an increased permeability leading to interstitial edema (25, 64, 130). Plasma mtDNA has also been associated with MODS as well as mortality in severe trauma (16).

### Late MODS

In addition to SIRS, as mentioned above, DAMPs may lead to an IS that is directly responsible for increased susceptibility to secondary infection, mainly hospital-acquired pneumonia (HAP). Pneumonia in the intensive care unit (ICU) threatens patient prognosis. Of note, after severe trauma or TBI, the HAP rate can reach 40% (8). HAP can give rise to ALI, acute respiratory distress syndrome, severe sepsis and even MODS (131, 132). As a result, HAP increases ventilation duration, length of ICU stays and worsens prognosis in ICU trauma patients (133).

### Disseminated Intravascular Coagulation

Disseminated intravascular coagulation is a frequent issue following severe trauma. DIC can alter microcirculation leading to MODS following severe trauma. Among DIC determinants, many DAMPs were reported to disturb the procoagulant-anticoagulant balance. For instance, the level of circulating serum HMGB1 or DNA–histone complex are two independent prognostic factors of DIC (134, 135). Specifically, nucleic acids and F-MIT activate coagulation pathways (73) whereas histones activate thrombin formation and platelets aggregation (136). In the same way, HMGB1 promotes coagulation by enhancing tissue factor expression on monocytes and neutrophil extracellular trap release (134). At the opposite, histones and HMGB1 inhibit Protein C activation and impaired coagulation (137, 138).

## Conclusion

**Figure 3:**
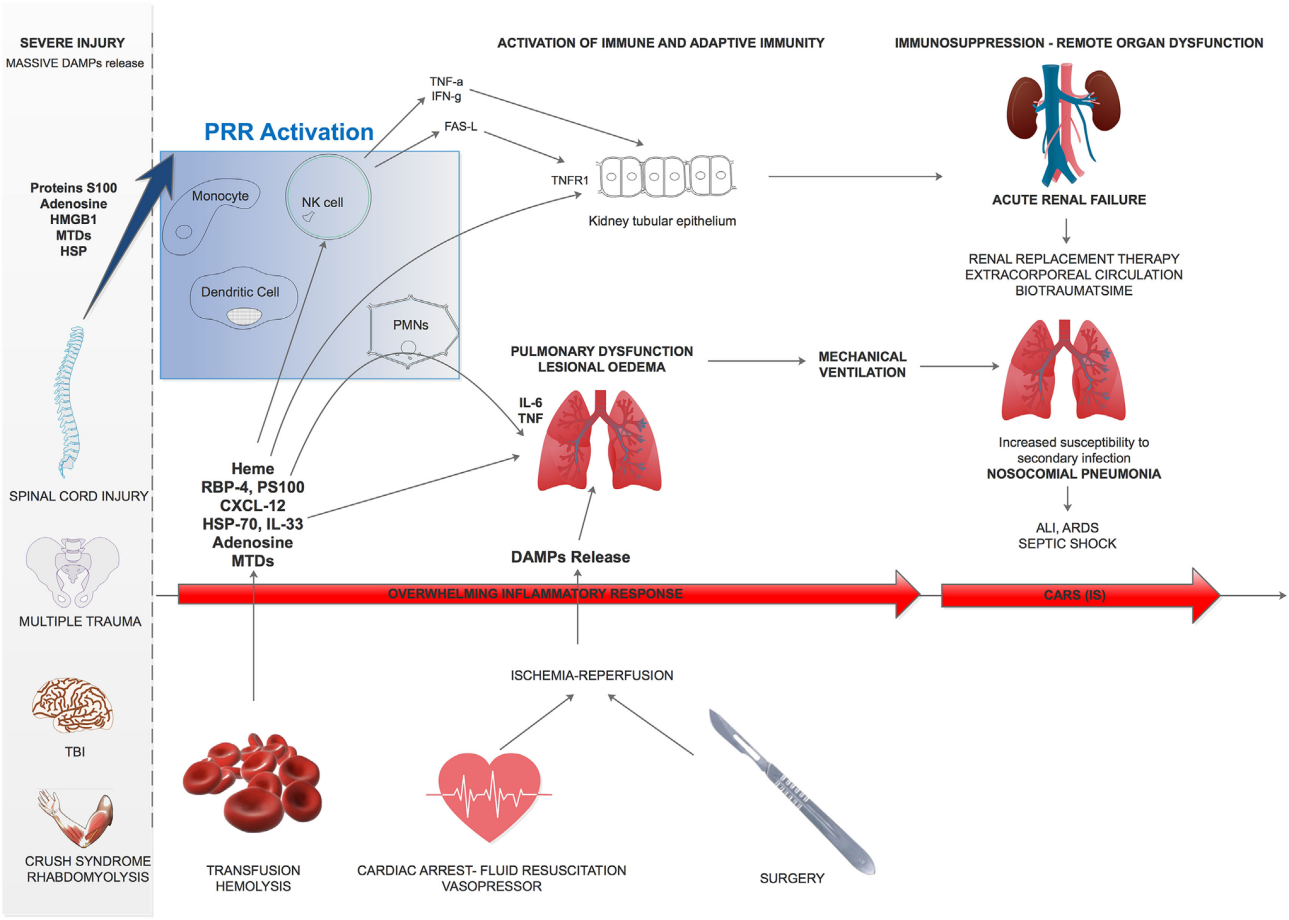
Damage-associated molecular patterns (DAMPs) effect overview on remote organ dysfunction after trauma. After severe trauma, multiple DAMPs are released and can trigger lung edema or kidney tubular epithelium activation. Besides, subsequent transfusion, fluid resuscitation and surgery will worsen these phenomenon and lead to remote acute organ failure. ALI, acute lung injury; ARDS, acute respiratory distress syndrome; CARS, compensatory anti-inflammatory response; PMN, polymorphonuclear leukocytes; MTD, mitochondrial damage-associated molecular patterns; RBP4, retinol-binding protein-4; PS100, protein S100; HSP, heat-shock protein; TNFR1, Type 1-TNF receptor; TNF-α, tumor necrosis factor-α; HMGB1, high-mobility group box 1.

In conclusion, during severe injury, DAMPs are responsible for multiple innate and adaptive immune subset activation (Figure 3). The early stage of trauma is characterized by an inflammatory response leading to organ failure whose intensity differs between patients. Five to 15 days after trauma, a CARS occurs, leading to IS and increased susceptibility to nosocomial infection. This immunosuppressive phenotype results from the multiple aspects of immunity impairment (Figure 3).

## Author Contributions

MV, AR, and KA wrote the main manuscript. All authors reviewed the manuscript.

## Conflict of Interest Statement

The authors declare that the research was conducted in the absence of any commercial or financial relationships that could be construed as a potential conflict of interest.

## Abbreviations

ALI, acute lung injury; APC, antigen-presenting cells; ARDS, acute respiratory distress syndrome; ATP, adenosine 5′-triphosphate; CARS, compensatory anti-inflammatory response syndrome; CXCL12, SDF1-α (stromal cell-derived factor 1); DAMPs, damage-associated molecular patterns; DIC, Disseminated intravascular coagulation; DNA, desoxynucleic acid; ERK, extracellular signal-regulated kinase; F-MIT, N-formyl peptides; FRP1, formyl peptide receptor-1; GPCR, G protein-coupled surface receptors; HAP, hospital-acquired pneumonia; HLA, human leukocyte antigen; HMGB1, high-mobility group box 1; HSP, heat-shock protein; ICU, intensive care unit; IKK, IκB kinase; IFN, interferon; IRAK, IL-1 receptor-associated kinase-4; IRF, interferon regulatory factor; IS, Immunosuppression; ISS, injury severity score; ITAM, immunoreceptor tyrosine-based activation motif; LPS, lipopolysaccharide; MD-2, myeloid differentiation factor 2; MHC, major histocompatibility complex; MMP9, metalloproteinase 9; MTD, mitochondrial damage-associated molecular patterns; mtDNA, mitochondrial DNA; MODS, multiple organ dysfunction syndrome; MRPs, myeloid-related proteins; MyD88, myeloid differentiation protein 88; NET, neutrophil extracellular trap; NK, natural killer; NLRP3, NOD-like receptors pyrin domain containing 3; NOD, nucleotide oligomerization domain; PD-1, program death-1; PI3K, phosphoinositide-3 kinase; PRR, pattern recognition receptor; PRBC, packed red blood cells; RAGE, receptor for advanced glycation end products; RBP4, retinol-binding protein 4; ROS, radical oxygen species; SIRS, systemic inflammatory response syndrome; TBI, traumatic brain injury; TREM-1, triggering receptor expressed on myeloid cells-1; TRIF, TIR domain-containing adaptor protein inducing interferon β.
