# Supplementary material for: Trauma-Induced Damage-Associated Molecular Patterns-Mediated Remote Organ Injury and Immunosuppression in the Acutely Ill Patient
Source: Front Immunol. 2018 Jun 15;9:1330. doi: 10.3389/fimmu.2018.01330 (PMC6013556; doi:10.3389/fimmu.2018.01330)
Supplement: Supplementary file 1 [file presentation_1.PDF]

TITLE PAGE : Trauma-induced, DAMP-mediated remote Organ injury and immunosuppression in the acutely ill patient, hosted by Dr julien Pottecher, Walter Gottlieb Land, Carl Jeffrey Hauser, Alain Meyer, Camilla Ferreira Wenceslau, Kim Timmermans, Frontiers in Immunology, Section Inflammation.

Mickael VOURC'H, MD, PhD (1,2), Antoine ROQUILLY MD PhD (1,2), Karim ASEHNOUNE, MD, PhD (1,2)*.

Affiliations

(1) Laboratoire UPRES EA3826 «Thérapeutiques cliniques et expérimentales des infections», IRS2 - Nantes Biotech, Université de Nantes, Nantes, France,

(2) Intensive Care Unit, Anesthesia and Critical Care Department, Hôtel Dieu, University Hospital of Nantes, Nantes, France

* Corresponding author

Online-only Material: Supplementary Figures, Video, Table and Legends

eFigure 1: Retinol-Binding protein-4 and CXCL-12 concentration in packed red blood cell supernatant

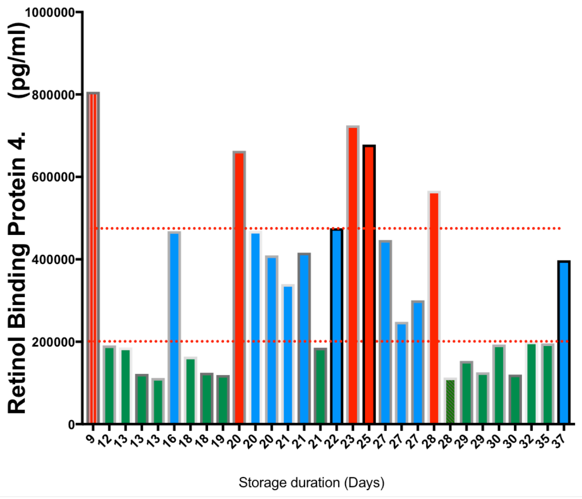

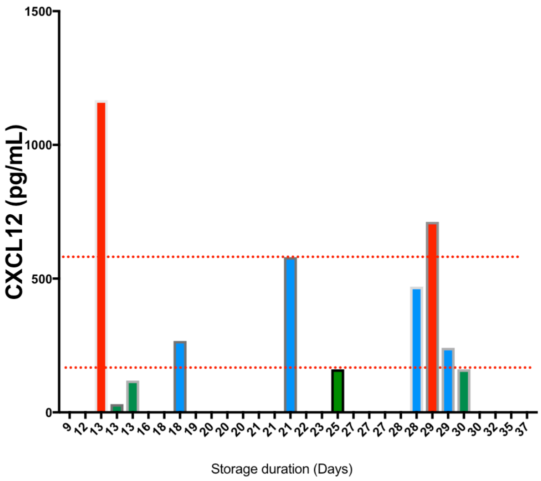

A **B**

Legends eFigure 1: Representative histograms of (**A**) Retinol Binding Protein-4 (RBP4) and (B) CXCL12 concentrations out of 30 distinct packed red blood cells (PRBC) according to the storage duration at the blood. Bank. The storage duration is mentioned below the horizontal axis in days. Green, Blue and red histograms stand for respectively low, moderate and high concentrations of proteins in PRBC supernatant. There was no correlation between storage duration and proteins concentrations.
